# New Myrtenal–Adamantane Conjugates Alleviate Alzheimer’s-Type Dementia in Rat Model

**DOI:** 10.3390/molecules27175456

**Published:** 2022-08-25

**Authors:** Stela Dragomanova, Maria Lazarova, Aldar Munkuev, Evgeniy Suslov, Konstantin Volcho, Nariman Salakhutdinov, Amina Bibi, Jóhannes Reynisson, Elina Tzvetanova, Albena Alexandrova, Almira Georgieva, Diamara Uzunova, Miroslava Stefanova, Reni Kalfin, Lyubka Tancheva

**Affiliations:** 1 Institute of Neurobiology, Bulgarian Academy of Sciences, Acad. G. Bonchev St., Block 23, 1113 Sofia, Bulgaria; 2 Department of Pharmacology, Toxicology, and Pharmacotherapy, Faculty of Pharmacy, Medical University, 9002 Varna, Bulgaria; 3 Department of Medicinal Chemistry, Novosibirsk Institute of Organic Chemistry of the Russian Academy of Sciences, Lavrentiev Av. 9, 630090 Novosibirsk, Russia; 4 School of Pharmacy and Bioengineering, Keele University, Hornbeam Building, Staffordshire ST5 5BG, UK; 5 Department of Healthcare, South-West University “Neofit Rilski”, Ivan Mihailov St. 66, 2700 Blagoevgrad, Bulgaria

**Keywords:** neuroprotection, experimental dementia, myrtenal–adamantane conjugates, acetylcholinesterase, monoamines, memory

## Abstract

Alzheimer’s disease (AD) is a neurodegenerative disease associated with memory impairment and other central nervous system (CNS) symptoms. Two myrtenal–adamantane conjugates (MACs) showed excellent CNS potential against Alzheimer’s models. Adamantane is a common pharmacophore for drug design, and myrtenal (M) demonstrated neuroprotective effects in our previous studies. The aim of this study is to evaluate the MACs’ neuroprotective properties in dementia. Methods: Scopolamine (Scop) was applied intraperitoneally in Wistar rats for 11 days, simultaneously with MACs or M as a referent, respectively. Brain acetylcholine esterase (AChE) activity, noradrenaline and serotonin levels, and oxidative brain status determination followed behavioral tests on memory abilities. Molecular descriptors and docking analyses for AChE activity center affinity were performed. Results: M derivatives have favorable physicochemical parameters to enter the CNS. Both MACs restored memory damaged by Scop, showing significant AChE-inhibitory activity in the cortex, in contrast to M, supported by the modeling analysis. Moderate antioxidant properties were manifested by glutathione elevation and catalase activity modulation. MACs also altered noradrenaline and serotonin content in the hippocampus. Conclusion: For the first time, neuroprotective properties of two MACs in a rat dementia model were observed. They were stronger than the natural M effects, which makes the substances promising candidates for AD treatment.

## 1. Introduction

Neurodegenerative diseases are a large group of pathological conditions—hereditary or sporadic—with a similar etiology. They are characterized by progressive loss of neuronal structures and functions, as well as neuronal death. The most common neurodegenerative condition is Alzheimer’s disease (AD), which is associated with changes in memory, thinking, orientation, and behavior [1]. The influence of oxidative stress on the occurrence of memory disorders and the free-radical nature of AD and dementias have been proven. The accumulation of toxic oxidation products in some brain structures over time affects neuronal function [2]. Neurodegeneration is associated with cognitive deficits and with a significant loss of cholinergic neurons. This usually occurs in brain regions responsible for memory and learning, such as the hippocampus, cortex, and basal nucleus of Meynert [3]. The cholinergic system, together with other mediator systems, forms a neuronal network in the dorsal hippocampus, which is crucial for learning and memory processes [4]. An imbalance between the cholinergic and serotonergic systems contributes to cognitive impairment in Alzheimer’s disease [5]. In addition, altered central nervous system (CNS) monoamine receptor density is observed in AD, making targeting specific subtypes of dopamine, serotonin, and norepinephrine receptors a potential therapeutic approach [6].

**Figure 1:**
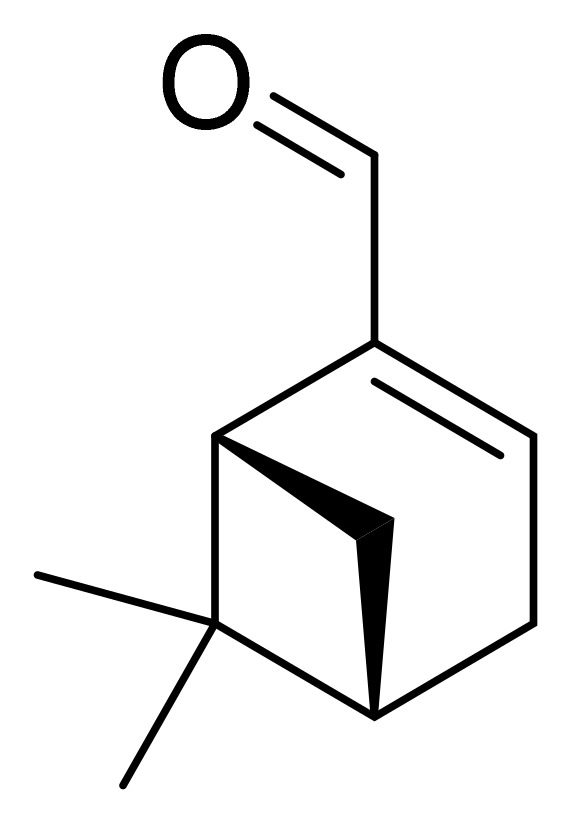
The structure of (-)-myrtenal.

Myrtenal (M) (Figure 1) is a bicyclic monoterpenoid of natural origin with biological activities, e.g., anti-acetylcholinesterase activity in vitro [7]. M has demonstrated in vivo neuroprotective effects in scopolamine-induced dementia, manifested by memory improvement, cholinergic mediation stimulation, and specific antioxidant effects in rats’ brains. A histopathological investigation found a significantly increased number of vital neurons in the rat cerebral cortex [8,9]. In another experiment, we discovered that M had beneficial effects in the 6-hydroxydopamine (6-OHDA)-induced experimental model of Parkinson’s disease, with positive effects on memory and coordination, reduced oxidative stress, and increased dopamine content in the lesioned cerebral hemisphere in rats [10].

M is a volatile, lipophilic compound, and therefore it is difficult to formulate emulsions containing it. Furthermore, monoterpenes and monoterpenoids often lack selectivity and suffer from low metabolic stability. Thus, chemical modification of a monoterpene can modulate its biological effects, including selectivity, by changing its physicochemical characteristics such as lipophilicity and volatility.

Adamantane is a well-established pharmacophore based on known pharmacological agents [11]. It is used for synthesis and development of medicines in various fields. Some of its derivatives are indicated for neurodegeneration treatment, namely memantine for AD and amantadine for Parkinson’s disease [12]. Moreover, the incorporation of an adamantane fragment could enhance permeation of the blood–brain barrier and improve metabolic stability without additional cytotoxicity.

**Figure 2:**
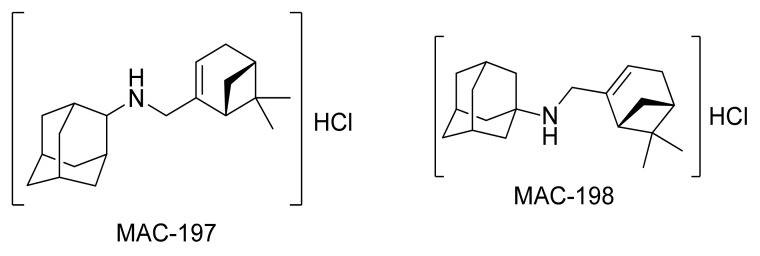
Chemical structure of MAC-197 and MAC-198.

Because M exerts neuroprotective properties and the adamantine structure is established for drug development, myrtenal–adamantane conjugates (MACs) have been synthesized as reported by Teplov et al. [13], followed by the preparation of hydrochlorides to study their neuroprotective potential (Figure 2). Previously, these compounds, in the form of free bases, demonstrated anxiolytic activity [14], which makes them potential modulators of CNS activity.

The aim of this study is to examine comparatively the neuroprotective potential of these two MACs in scopolamine-induced Alzheimer’s-type dementia in laboratory rats.

## 2. Results

### 2.1. Physicochemical Propertied Affecting Blood-Brain Barrier Diffusion

The calculated molecular descriptors MW (molecular weight, g/mol), log *p* (water-octanol partition coefficient), HD (hydrogen bond donors), HA (hydrogen bond acceptors), PSA (polar surface area, Å^2^), and RB (rotatable bonds) of the ligands are given in Table 1. The values for the molecular descriptors all lie within lead-like chemical space except for log *p*; MAC-197 and MAC-198 have relatively high log *p* values in the upper end of drug-like chemical space due to their adamantine moiety (for the definition of lead-like space, drug-like space, and Known Drug Space regions, see Zhu et al. (2012) [15] and Table S1). The log *p* values show the enhanced lipophilicity of the conjugates compared to the natural product M.

To glean further insight into the effects of the molecular descriptors used, 208 compounds with experimentally established blood–brain barrier (BBB) permeability were collected [16,17,18,19]. These values are defined as the logarithmic ratio between the concentrations in the brain(*c_Brain_*) and in the blood (*c_Blood_*); see Equation (1).

(1) $$\log BB=log\left(\frac{c_{Brain}}{c_{Blood}}\right)$$

**Figure 3:**
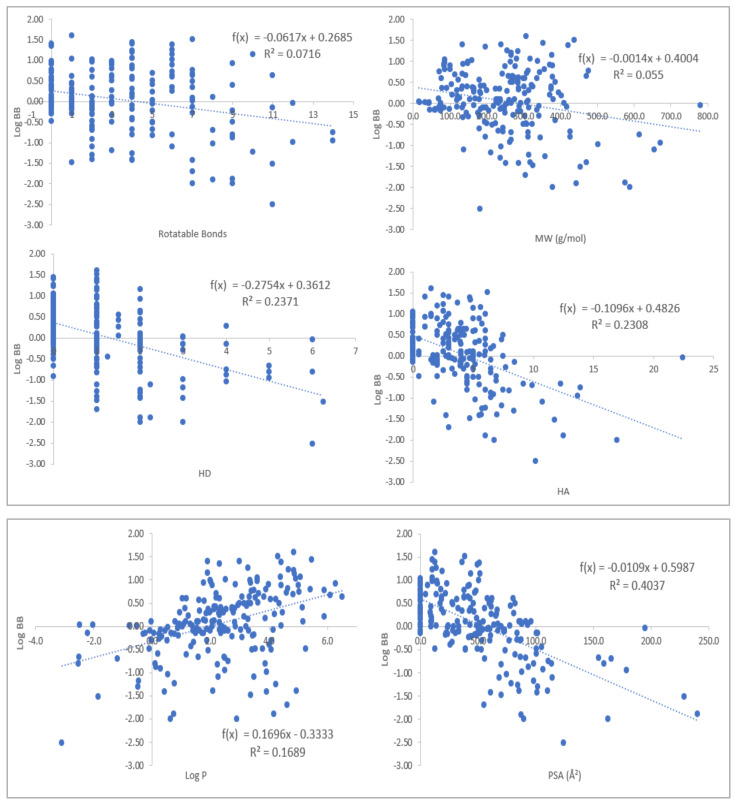
The correlations of the molecular descriptors with experimentally derived log BB values (*n* = 208). Higher values of HD, HA, and PSA impede permeability through the barrier, but higher values of log *p* facilitate it.

The values were correlated with the molecular descriptors; the results are shown in Figure 3, and the numerical values are given in Table S2.

When the correlations and trends in Figure 3 are considered and compared to the values of MAC-197 and MAC-198, the ligands are in excellent property space. On the one hand, they both have relatively high log *p* values, and on the other hand, they have low HD, HA, and PSA values, i.e., the optimal position for BBB permeability.

The Known Drug Indexes (KDIs) for the ligands were calculated to gauge the balance of the molecular descriptors (MW, log *p*, HD, HA, PSA, and RB). This method is based on the analysis of drugs in clinical use, i.e., the statistical distribution of each descriptor is fitted to a Gaussian function and normalized to 1, resulting in a weighted index. Both the summation of the indexes (KDI_2a_) and multiplication (KDI_2b_) methods were used [20], as shown for KDI_2a_ in Equation (2) and for KDI_2b_ in Equation (3); the numerical results are given in Table 1.

KDI_2a_ = I_MW_ + I_log *p*_ + I_HD_+ I_HA_ + I_RB_ + I_PSA_ (2)

KDI_2b_ = I_MW_ × I_log *p*_ × I_HD_× I_HA_ × I_RB_ × I_PSA_ (3)

The KDI_2a_ values for the ligands are 4.05 (MAC-197), 3.89 (MAC-198), and 3.38 for M, with a theoretical maximum of 6 and an average of 4.08 (±1.27) for known drugs. The drug collection of 1880 entries from Eurtivong and Reynisson (2018) was analyzed [20], and 245 drugs have an indication for diseases in the CNS (see a complete list in Table S3). The average is 4.54 (±0.67) for the CNS-active pharmaceuticals, slightly higher than for MAC-197 and MAC-198. KDI_2b_ has values of 0.07 (MAC-197), 0.04 (MAC-198), and 0.01 for M, with a theoretical maximum of 1 and with a KDS average of 0.18 (±0.20); the CNS-active drugs have an average of 0.21 (±0.19); KDI_2b_ is very unforgiving, as the parameters are multiplied, and a single low number results in a low overall value. It can therefore be concluded that the M derivatives have favorable physicochemical parameters to enter the CNS.

### 2.2. Effects of MACs on Scopolamine-Impaired Memory in Rats

**Figure 4:**
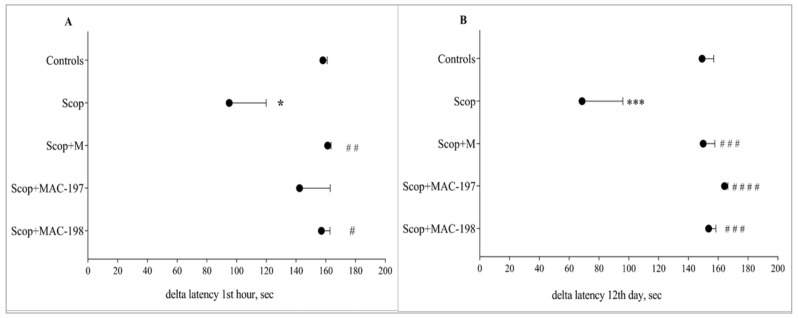
Effects of MAC-197 and MAC-198 on short-term (**A**) and long-term (**B**) memory performance in rats with scopolamine-induced dementia; M (40 mg/kg) was used as a referent; data are expressed as the mean ± SEM (*n* = 10). * *p* < 0.05, *** *p* < 0.001 vs. Controls; ^#^ *p* < 0.05, ^# #^ *p* < 0.01, ^# # #^ *p* < 0.001, ^# # # #^ *p* < 0.0001 vs. scopolamine (Scop) group.

Scopolamine (Scop) induced a significant impairment in both short-term and long-term memory, as expected. In acute treatment, Scop reduced the delta latency by 39.9% (*p* < 0.05) (Figure 4A), and after multiple applications, by 54% (*p* < 0.001) (Figure 4B), as compared to the Controls group.

M restored the rats’ memory to a similar lever as the Controls (*p* < 0.01) after acute treatment with parameter elevation by 69.7% (Figure 4A) and 118.4% (*p* < 0.001) on day 12 (Figure 4B) when compared to Scop, as seen in our previous studies [8].

Both MAC groups responded similarly to M in demented animals. However, MAC-197 showed a better effect on Scop-damaged long-term memory—the increase in the indicator in comparison to Scop-treated rats was 139.3% (*p* < 0.0001, Figure 4B). MAC-198 restored both short- and long-term memory—the delta latency was increased by 65.3% (*p* < 0.05) and 123.8% (*p* < 0.001), respectively, as compared to the Scop group.

### 2.3. Effects of MACs on Brain AChE Activity

#### 2.3.1. Docking Study

The predicted MACs’ affinity to the AChE enzyme catalytic center is shown in Table 2.

For further comparison, acetylcholine, tacrine, and donepezil were docked into the binding site. The two latter compounds are acetocholinesterase inhibitors that see clinical use for AD. According to all four scoring functions (Table 2), both MAC-197 and MAC-198 have stronger interaction with the AChE binding pocket than both M and acetylcholine; the MACs have somewhat lower predicted affinity than the drugs.

**Figure 5:**
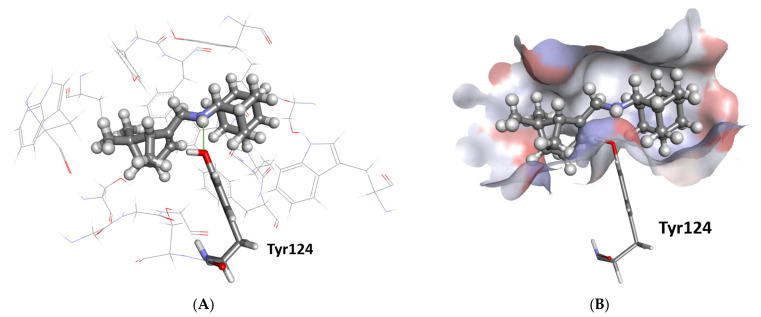
The docked pose of MAC-197 in the binding site of AChE as predicted by the ChemPLP scoring function. (**A**) The predicted configuration is shown in the ball-and-stick format, and the hydrogen bonding to Tyr124 (stick format) is shown as a green line. The amino acids forming the lipophilic binding pocket are Trp86, Gly120, Gly121, Gly122, Trp286, Val294, Phe295, Phe297, Tyr337, Phe338, Tyr341, and His447; they are shown as lines. (**B**) The protein surface is rendered; blue depicts regions with a partial positive charge on the surface; red depicts regions with a partial negative charge, and grey shows neutral areas. The ligand occupies the deep binding pocket.

As predicted by ChemPLP, MAC-197 forms one hydrogen bond with the hydroxyl oxygen atom in Tyr124 for AChE (Figure 5A). The myrtenyl moiety is pushed deep into the binding pocket, with the adamantane at the entrance (Figure 5B). The pocket is made up of many lipophilic amino acid residues accommodating the two aliphatic components of the ligand. Interestingly, MAC-198 is predicted to have the reverse binding conformation, i.e., the myrtenyl moiety is at the entrance of the binding pocket. Finally, M is predicted to occupy the bottom of the binding site, the same site of the myrtenyl moiety for MAC-197.

Based on the modeling, AChE is a plausible target for the substances tested, with the ligands forming hydrogen bonds and fitting well into the binding pocket without straining the molecular conformation into energetically unfavorable conformations.

#### 2.3.2. Evaluation of Brain AChE Activity In Vivo

**Figure 6:**
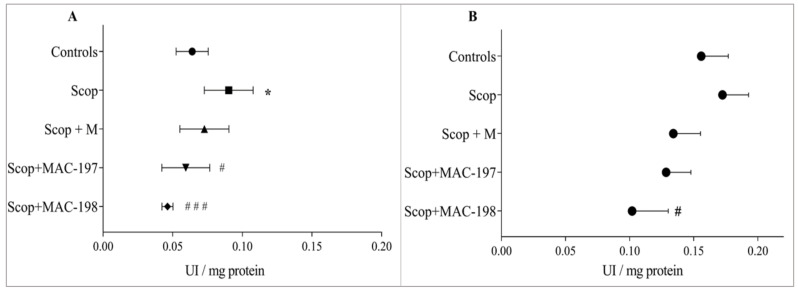
Effects of MAC-197 and MAC-198 on brain AChE activity in the cortex (**A**) and the hippocampus (**B**) in rats with scopolamine-induced dementia; M (40 mg/kg) was used as referent; UI—international units; data are expressed as the mean ± SEM (*n* = 5). * *p* < 0.05 vs. Controls; ^#^ *p* < 0.05, ^# # #^ *p* < 0.001 vs. Scop group.

Scop produced an increase in AChE activity significantly in the cortex (41%, *p* < 0.05, Figure 6A) and in the hippocampus (10.6%, *p* = n.s., Figure 6B) as compared to the Controls.

In the cerebral cortex, both MAC derivatives showed significant AChE-inhibitory activity (Figure 6A) in comparison to natural M, which did not significantly affect brain AChE activity. The enzyme activity was reduced by 34.2% (*p* < 0.05) by MAC-197 and by 48.7% (*p* < 0.001) by MAC-198 as compared to Scop-treated animals.

In the hippocampus, both M and MAC-197 showed no significant tendency for AChE-inhibitory activity. The most pronounced decrease of 41% was caused by MAC-198 (*p* < 0.05, Figure 6B).

### 2.4. Effect of MACs on Brain Noradrenaline and Serotonin Levels

#### 2.4.1. Noradrenaline Levels

**Figure 7:**
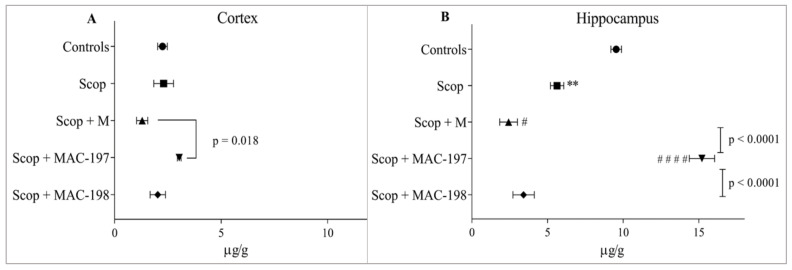
Effects of MAC-197 and MAC-198 on brain NA levels in cortex (**A**) and hippocampus (**B**) in rats with scopolamine-induced dementia; M (40 mg/kg) was used as a referent; data are expressed as the mean ± SEM (*n* = 5). ** *p* < 0.01 vs. Controls; ^#^ *p* < 0.05, ^# # # #^ *p* < 0.0001 vs. Scop group.

Scop did not affect noradrenaline (NA) cortical content (Figure 7A) but induced a significant decrease in hippocampal NA level by 41% (*p* < 0.01, Figure 7B).

In the cortex of M-treated rats, NA content was reduced as compared to the Scop group (*p* = n.s.), as MAC-197 increased the indicator by 134.3% compared to the Scop + M group (*p* = 0.018) and by 35% compared to the Controls (Figure 7A).

In the hippocampus, application of M and MAC-198 resulted in an even greater decrease in mediator levels, even from the scopolamine group, of 57% (*p* < 0.05) for M and 39.4% for MAC-198 (*p* = n.s., Figure 7B). Only MAC-197 elevated NA content, doing so by 170% as compared to the Scop group (*p* < 0.0001) and by 59.4% in comparison to Controls.

#### 2.4.2. Serotonin Levels

**Figure 8:**
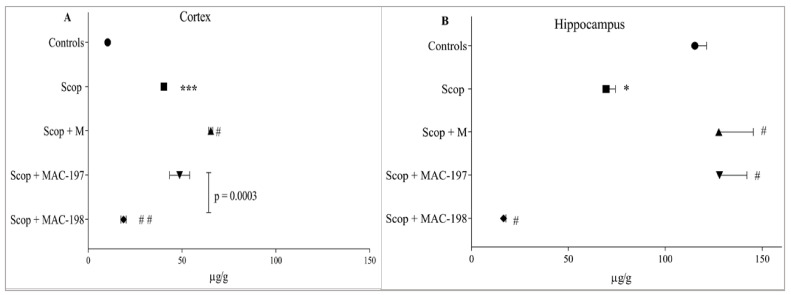
Effects of MAC-197 and MAC-198 on brain 5-HT levels in the cortex (**A**) and the hippocampus (**B**) in rats with scopolamine-induced dementia; M (40 mg/kg) was used as a referent; data are expressed as the mean ± SEM (*n* = 5). * *p* < 0.05, *** *p* < 0.001 vs. Controls; ^#^ *p* < 0.05, ^# #^ *p* < 0.01 vs. Scop group.

Serotonin (5-HT) content measured in the hippocampus was 10 times greater than in the cortex of the Controls group. Scop treatment led to a three-fold increase in mediator levels in the cortex (*p* < 0.001, Figure 8A), while in the hippocampus, the indicator was decreased by 39.8% as compared to Controls (*p* < 0.05, Figure 8B).

In the cerebral cortex, M treatment significantly increased 5-HT content by 62% in comparison to the Scop group (*p* < 0.05, Figure 8A). The effect of MAC-197 on this indicator was close to the Scop + M group. MAC-198 had the opposite effect, lowering the mediator content by 53.4% compared to the Scop group (*p* < 0.01), with an average value close to that of the Controls.

In the hippocampus, M and MAC-197 significantly increased serotonin levels (*p* < 0.05 vs. Scop) (as observed in the cortex) by 83.8% and 84.4%, respectively, reaching the control level (Figure 8B). Again, MAC-198 exhibited the opposite effect by decreasing 5-HT content by 76.2% compared to Scop (*p* < 0.05).

### 2.5. Antioxidant Activity of MACs in the Brain Cortex

**Figure 9:**
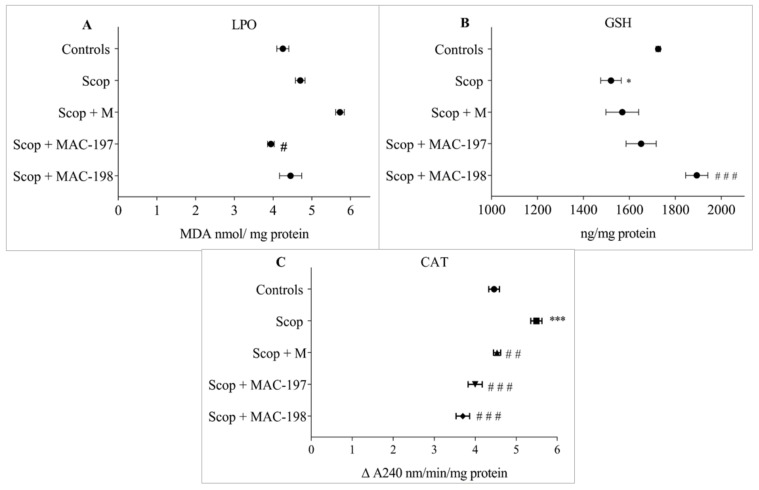
Antioxidant effects of MAC-197 and MAC-198 in brain cortex in rats with scopolamine-induced dementia—lipid peroxidation (LPO) products level (**A**), GSH content (**B**), and CAT activity (**C**); M (40 mg/kg) was used as a referent; data are expressed as the mean ± SEM (n = 5). * *p* < 0.05, *** *p* < 0.001 vs. Controls; ^#^ *p* < 0.05, ^# #^ *p*< 0.01, ^# # #^ *p* < 0.001 vs. Scop group.

The Scop treatment induced oxidative stress in the cerebral cortex, as expected. It was manifested by an elevation in lipid peroxidation (LPO) products levels of 10.6% (*p* = n.s., Figure 9A) and a GSH content reduction of 11.9% (*p* < 0.05) as compared to Controls (Figure 9B). Catalase activity was significantly increased by 23.1% (*p* < 0.001) (Figure 9C).

M insignificantly increased Malone dialdehyde (MDA) concentration compared to the Scop group (Figure 9A). However, its specific antioxidant properties were manifested by restoration of the elevated-by-scopolamine CAT activity to close to the control level—by 17.5% (*p* < 0.01) vs. the Scop group (Figure 9C).

The MACs significantly affected the parameters for oxidative status. MAC-197 decreased the LPO products level by 16.1% (*p* < 0.05, Figure 9A), and MAC-198 increased the GSH content by 24.6% (*p* < 0.001, Figure 9B) compared to Scop. Both substances decreased the elevated-by-scopolamine-application CAT activity—by 27.2% for MAC-197 and by 32.6% for MAC-198 (*p* < 0.001, Figure 9C).

## 3. Discussion

Neurodegenerative disorders are complex diseases characterized by multifactorial pathoetiology [21]. Nowadays, research is focused on the discovery of new therapeutic agents with more than one target for pharmacological action. This is based on the understanding that innovative, multi-purpose ligands can more effectively counteract the complexity of pathological processes in neurodegenerative diseases to overcome the challenges of the polypharmacological approach [22]. The search for potential pharmacological agents with complex mechanisms of action includes different substances, most of them being natural compounds [23,24,25].

The ameliorative activity of M in conditions of scopolamine-induced AD-type dementia and the 6-OHDA-induced experimental model of Parkinson’s disease has been already demonstrated in our previous research [9,10]. Our results revealed its complex neuroprotective mechanisms of action, which are beneficial to the memory of rodents, neuromodulatory, and antioxidant, as supported by histopathological examinations.

A literature review found that adamantane derivatives effectively modulate neurodegenerative and inflammatory processes via different mechanisms. Some of the pharmacological targets are clear—they act on AMPA and KATP channels, GABA, and serotonin receptors [26]. One of the most important properties of the adamantane-derived compounds is their ability to penetrate through the blood–brain barrier (BBB), which is a key property of a potential neuroprotective drug. Hydroxamic acids with adamantane scaffolds restored normal memory functions in 5xFAD mice that simulated AD to the level observed in control wild-type animals [27].

The combination of a monoterpene moiety with an adamantane fragment showed enhanced biological activity, improved selectivity, and increased metabolic stability. For example, SQ109 is a novel anti-tubercular agent combining adamantane and geranyl scaffolds, which is currently in phase II clinical trials [28]. Compounds consisting of adamantane and monoterpenoid fragments were shown to inhibit tyrosyl-DNA-phosphodiesterase 1 (Tdp1), a DNA repair enzyme which is considered an important target for increasing the efficacy of topoisomerase poisons [29]. The two conjugates investigated in this study were designed and synthesized to avoid some M drawbacks and to increase the biological efficacy of natural M. Like most essential oil natural components, M is a volatile compound. In this regard, linkage with an adamantane moiety increased the lipophilicity of the conjugates and eliminated M’s volatility. Based on the results presented here, it can be stated that M derivatives have favorable physicochemical parameters to penetrate the BBB, influencing CNS functions. As M has a complex neuroprotective effect, it can be assumed that its derivatives would also show similar or better properties.

MAC-197 and MAC-198 were reported to exhibit antiviral activity against influenza virus A (H1N1)pdm09 [13], anticancer effects [30], and anxiolytic activity [14]. To the best of our knowledge, there are no data in the literature on the study of their neuroprotective properties. In the current research, new MAC derivatives were evaluated for neuroprotective activity in Scop-induced dementia of the AD type. As a muscarinic antagonist, Scop is widely used to initiate experimental dementia of the AD type [31,32,33,34]. Its intraperitoneal administration causes dysregulation of the brain’s cholinergic system with these consequences: increased AChE activity, oxidative stress, and decreased levels of ACh [9,35,36]. On the behavioral level, these biochemical changes are revealed as a pronounced cognitive deficit in the experimental animals. The brain’s biogenic amine levels are also reduced after Scop administration. This is in agreement with our previous work [37], as well as reports by other researchers [38,39,40,41]. In this way, the model repeats some of the main characteristics of AD [42].

The biological activity of MACs in the Scop-induced model of dementia was conducted by behavioral observation and biochemically; the effects were compared with those of M. The molecular modeling predicted MACs’ affinity to bind to the active center of AChE. Their effects on memory, AChE activity, NA and 5-HT content, as well as antioxidant capacity in the rats’ brains, were established.

It is well known that the first clinical symptoms in AD patients are associated with memory deficits [43]. The effects of MACs on short- and long-term learning and memory performance in the rats, compared to those of M, were evaluated by a passive avoidance test. This is a fear-aggravated test, where the difference in latency time of the reaction vs. initial latency is taken as a memory status indicator. On the one hand, it was found that the M and MAC-198 alleviated the detrimental effect of Scop dosing for both short- and long-term memory performance in the animals. On the other hand, MAC-197 application induced interesting behavior, with a less pronounced positive effect on memory after a single treatment compared to M and MAC-198, and exceeding them after repeated application.

Normal functioning of central cholinergic transmission is essential in the regulation of memory and mood [44]. AD is characterized by cholinergic dysfunction, manifested by increased AChE activity and decreased ACh content in the brain [42]. The scopolamine model of dementia causes similar changes in these indicators. Concerning AChE, unlike M, both MACs demonstrated an AChE-inhibitory effect, which was most pronounced for MAC-198. This effect was observed in both the cortex and the hippocampus and supported by our molecular modeling results showing a stronger affinity of MACs to the active site of the enzyme compared to that of M.

Along with the well-known cognitive symptoms of the disease, AD is also accompanied by non-cognitive ones, such as depression, anxiety, agitation, eating and sleeping disorders, and aggression [45]. Cognitive and non-cognitive symptoms of the disease are due to modulated levels of biogenic amines in patients’ brains. Gutierrez et al. summarized the available information on the role of norepinephrine levels in cognitive dysfunction and the progression of neurodegenerative processes [46].

In the present study, Scop treatment produced a significant decrease in hippocampal NA content (*p* < 0.01), in line with the research work of Falsafi et al. and Garcia-Alloza et al. [5,47]. On the one hand, M and MAC-198 did not reverse decreased NA levels in either the cortex or the hippocampus. Furthermore, in the hippocampus, they potentiated the effect of Scop. On the other hand, MAC-197 increased NA content in the cortex (ns) and the hippocampus (*p* < 0.01 vs. Scop), revealing its antidepressant potential. In our opinion, this difference in the two new compounds‘ effects probably can be found in the chemical structures of the two derivatives, as the myrtenyl compound is attached via an amino group to the first and second position, respectively, in the adamantane molecule.

Serotonin levels are essential not only for the occurrence of depression but also in behavioral changes in AD. Its role together with acetylcholine in cognitive problems in neurodegenerative processes has also been clarified [48]. An imbalance between cholinergic and serotonergic systems contributes to cognitive impairment in AD [5]. AD has been associated with the loss of serotonergic neurons and a reduction in the levels of 5-hydroxytryptamine, as seen in the postmortem brains of patients with this disease [49,50].

In the current research, Scop application induced a significant increase in 5-HT content in the cortex (*p* < 0.001) and a significant decrease in the hippocampus (*p* < 0.05), with mediator control concentrations 10 times higher in the hippocampus. These results correlate with previous studies, according to which serotonin levels are different in some brain areas, and they depend on the age of the rats [51].

All tested substances changed 5-HT levels in both brain structures related to the memory—the cortex and hippocampus. MAC-198 decreased its level, but M and MAC-197 increased it. The effects of M and MAC-197 were accompanied by one interesting feature: in the cortex, they potentiated the effect of Scop, and in the hippocampus, they reversed it. The opposite effects of MAC-197 and MAC-198 are probably connected also to chemical structure differences in the two derivatives.

Increased levels of oxidative stress parameters in the brains of AD patients are very well-documented [52,53,54,55,56], and this is one of the most popular hypotheses for AD pathogenesis [57].

The results of a number of studies have shown that oxidative stress is associated with memory dysfunction in the Scop-induced animal model of dementia [58,59,60,61]. In our experiment, which was focused on oxidative stress parameters in the cerebral cortex, Scop induced oxidative stress, as established by other authors [62,63,64,65]. It was manifested with a significant decrease in GSH content, increased CAT activity, and a tendency to increase the levels of lipid peroxidation products.

M, like most natural terpenoids, exhibits antioxidant activity, which has been found in a rat hepatocellular carcinoma model [66], as well as in a model of dementia in our previous studies [9]. In the conditions of this experiment, M’s specific antioxidant activity in demented rats was expressed by a significant decrease in CAT activity close to the Controls level (*p* < 0.01 vs. Scop). Both MACs showed antioxidant capacity. MAC-197 significantly decreased lipid peroxidation product content by 16.1% compared to the Scop group, and the most pronounced was the effect of MAC-198 on the elevation of GSH (24.6%) in comparison to Scop, with a level of significance of *p* < 0.001. Both tested substances showed better antioxidant properties in the brain cortex of demented rats than natural M.

## 4. Materials and Methods

### 4.1. Chemicals

Scopolamine (Lot: A0354964) and (-)-myrtenal 98% (Lot: A0363097) were purchased from ACRÔS Organics. The (-)-myrtenal (≥97%) used for chemical synthesis was purchased from Sigma Aldrich (St. Louis, MO, USA), while 1- and 2-aminoadamantane hydrochlorides were purchased from ACRÔS Organics (Geel, Belgium). MACs were synthesized in the Department of Medicinal Chemistry of Novosibirsk Institute of Organic Chemistry (Novosibirsk, Russia) in the accordance with the procedure of [13] by reaction of (-)-myrtenal with 1- or 2-aminoadamantane, followed by NaBH_4_ reduction of the corresponding imines. The spectral data were consistent with those previously reported [13]. NMR spectra are given in the Supplementary Materials (Figures S1–S4). The amines obtained were transformed into corresponding hydrochlorides by bubbling gaseous HCl through an ethereal solution of the amines, followed by filtration of the precipitate formed.

### 4.2. Modelling and Screening

The Scigress version FJ 2.6 program (Scigress Ultra V. F.J 2.6. 2016, Krakow, Poland) was used to build the inhibitors, and the MM3 [67,68,69] force field was applied to identify the global minimum using the CONFLEX method [70], followed by structural optimization.

The docking center for the AChE crystal structure (PDB ID: 5HF9, resolution 2.20 Å, Homo sapiens) was defined as the position of the nitrogen in the pyridine ring substituted with the oxime of the co-crystallized ligand 4-(aminocarbonyl)-1-[({2-[(*E*)-(hydroxyimino)methyl]pyridiunum-1-yl}methoxy) methyl]pydidinium (HI6) (x = 12.350, y = −55.749, z = −24.154) [71]. Fifty docking runs were allowed for each ligand using very flexible search efficiency (200%) with a 10 Å radius. The basic amino acids lysine and arginine were defined as protonated. Furthermore, aspartic and glutamic acids were assumed to be deprotonated. The GoldScore (GS) [72], ChemScore (CS) [73,74], ChemPLP (Piecewise Linear Potential) [75], and ASP (Astex Statistical Potential) [76] scoring functions were implemented to predict the binding modes and relative energies of the ligands using the GOLD v5.4.1 software suite (Cambridge, UK). It was also used to prepare the crystal structure for docking, i.e., the hydrogen atoms were added, while the co-crystallized ligands were removed, as were crystallographic water molecules. The co-crystallized HI6 ligand was re-docked into its binding sites to establish the prediction power of the GOLD docking software. The root-mean-square deviations (RMSD) were derived, i.e., the co-crystallized and docked conformations were overlain, and the distance of their heavy atoms was measured; the lower the number, the better the prediction, with RMSD < 1.0 Å considered good. The best scoring function for AChE is ChemPLP, with 1.896 Å, followed by ASP (8.423 Å), CS (6.937 Å), and GS (3.705 Å).

The QikProp 6.2 (2009) software(Schrödinger: New York, NY, USA) package was used to calculate the molecular descriptors. The reliability of QikProp was established for the calculated descriptors [77]. The Known Drug Indexes (KDI) were calculated from the molecular descriptors as described by Eurtivong and Reynisson (2018) [14]. For application in Excel, columns for each property were created, and the following equations were used to derive the KDI numbers for each descriptor: KDI MW: =EXP(−((MW−371.76)^2)/(2*(112.76^2))), KDI Log P: =EXP(−((LogP−2.82)^2)/(2*(2.21^2))), KDI HD: =EXP(−((HD−1.88)^2)/(2*(1.7^2))), KDI HA: =EXP(−((HA−5.72)^2)/(2*(2.86^2))), KDI RB =EXP(−((RB−4.44)^2)/(2*(3.55^2))), and KDI PSA: =EXP(−((PSA−79.4)^2)/(2*(54.16^2))). These equations could simply be copied into Excel and the descriptor name (e.g., MW) substituted with the value in the relevant column. To derive KDI_2A_, this equation was used: =(KDI MW + KDI LogP + KDI HD + KDI HA + KDI RB + KDI PSA). For KDI_2B_, this equation was used: =(KDI MW × KDI LogP × KDI HD × KDI HA × KDI RB × KDI PSA).

### 4.3. Experimental Rodents

The experiments were carried out on male adult Wistar rats (180 ÷ 220 g). The animals were kept under standard laboratory conditions in plastic cells, with a 12-h light/dark cycle, drinking water and food for rodents ad libitum, and optimal temperature, humidity, and indoor ventilation. The experimental protocols were carried out following the rules of the Committee on Ethics of the Bulgarian Food Safety Agency and in compliance with national laws and rules (Ordinance No. 20 of 01.11.2012 on the minimum requirements for the protection and welfare of experimental animals and requirements to establishments for their use, rearing and/or delivery, effective from 1 January 2013, issued by the Ministry of Agriculture and Food, Prom. SG issue 87 of 9 November 2012), based on the European Directive and in accordance with the rules of the Ethics Committee of the Institute of Neurobiology at the Bulgarian Academy of Sciences.

### 4.4. Experimental Design and Drug Treatment

Male adult Wistar rats were separated into five groups (*n* = 10): (1) Controls (Saline), (2) Scopolamine (Scop), (3) Scopolamine and Myrtenal (Scop + M), (4) Scopolamine and MAC-197 (Scop + MAC-197), and 5) Scopolamine and MAC-198 (Scop + MAC-198).

The scopolamine neurodegeneration model was induced via intraperitoneal application of the neurotoxic agent (1.0 mg/kg b.wt.) for 11 consecutive days as a solution of scopolamine hydrobromide dissolved in distilled water. The other substances were applied simultaneously with scopolamine in different intraperitoneal inoculations. The two myrtenal–adamantane conjugates (MACs) with code names MAC-197 and MAC-198 were injected in an effective dose of 1 mg/kg b.wt. M was used as a referent in an effective dose of 40 mg/kg b.wt., as established in our previous work [2]. M, as well as MACs, were injected as emulsions. All the solutions were prepared ex tempore and injected at the same time each day.

All animal groups were submitted to a behavioral test for memory ability status (passive avoidance test). The animals were then euthanized via mild CO_2_ inhalation. Their brains were quickly removed, and two main brain structures related to memory—the cortex and hippocampus—were separated for biochemical analysis. Brain acetylcholinesterase (AChE) activity, noradrenaline and serotonin levels, and oxidative status parameters were evaluated.

### 4.5. Behavioral Test

The step-through/passive avoidance test [78] was used to determine the state of both short-term and long-term memory [79]. In this protocol, to avoid electric current in the feet, the rodent must learn to stay in the brightly lit compartment of the apparatus and not enter the preferred dark compartment.

Initial latency (IL) was conducted before the treatment with the tested substances. Each animal was placed in the light half of the staging, leaving the door between the light and the dark part open. When a rat entered the dark (with four paws), the door closed, and a weak electrical shock was released through the floor (0.7 mA for 3 s).

The experimental phase included first testing 1 h after the first application and final testing at 24 h after the last one (on day 12); no electrical current was applied again. The latency time (up to 180 s), i.e., the time after which the animal moved into the dark room, was measured. As a memory status indicator, the difference in latency time vs. IL was recorded.

### 4.6. Biochemical Studies

#### 4.6.1. Evaluation of Brain AChE Activity

AChE activity in the cortex and hippocampus was determined according to the Ellman protocol [80]. Brain supernatants were added to a solution containing 1.0 mM acetyl thiocholine (AcSCh), 0.1 mM 5,5′-dithio-bis (2-nitrobenzoic acid) (DTNB), and 100 mM phosphate buffer (pH 8.0) and incubated for 5 min at 37 °C. The appearance of yellow color in the reaction of thiocholine with DTNB was spectrophotometrically detected (λ = 412 nm). The results are expressed as AChE µmol min/g protein.

#### 4.6.2. Evaluation of Noradrenaline and Serotonin Levels in Cerebral Cortex and HippoCampus

The concentration of the monoamines noradrenalin (NA) and serotonin (5-HT) in the brain cortex and hippocampus were measured via fluorescence reaction by the methods of Jacobowitz and Richardson [81]. Noradrenaline was extracted into the phosphate buffer and 5-HT into 0.1 N HCl. For NA fluorescence, the reaction requires ethylenediaminetetraacetic acid (EDTA), iodide solution, alkaline sulfite, and 5N CH_3_COOH, whereas for 5-HT, *o*-phthaldehyde must be added. Monoamines were determined at λ = 385/485 nm for NA and λ = 360/470 nm for serotonin, calculated based upon standard solution fluorescence, and expressed as µg/g of fresh tissue.

#### 4.6.3. Evaluation of Cerebral Cortex Antioxidant Activity

The following biochemical parameters were measured: total glutathione (GSH), measured according to Tietze (expressed as ng/mg protein) and with oxidized glutathione (GSSG) as a reference standard [82]. Lipid peroxidation product content was determined by the amount of thiobarbituric acid reactive substances (TBARs), expressed as nmoles Malone dialdehyde (MDA) per mg protein, with a molar extinction coefficient of 1.56 × 105 M^−1^cm^−1^ [83]. The post-nuclear homogenates of the brain structures (mg protein/mL) in 0.15 M KClmM potassium phosphate buffer, pH 7.4, were heated for 15 min at 100 °C in the presence of 2.8% tri-chloroacetic acid + 5N HCl + 2% thiobarbituric acid in 50 mM NaOH (2:1:1 *v*/*v*) for color developing. The absorbance was read at 532 nm against an appropriate blank.

Catalase (CAT) activity was determined according to Aebi [84]. The enzyme activity was expressed as ∆ E240/min/mg protein. Cu, Zn-superoxide dismutase (SOD) activity was determined according to Beauchamp and Fridovich [85], expressed as U/mg protein (one unit of SOD activity is the amount of the enzyme producing a 50% inhibition of Nitroblue tetrazolium reduction). Glutathione peroxidase (GPx) activity was measured by the method of Günzler et al. [86] and was expressed as nmol NADPH oxidized per minute per mg protein, with a molar extinction coefficient of 6.22 × 106 M^−1^cm^−1^.

Protein content was measured by the method of Lowry et al. [87].

### 4.7. Statistical Analysis

Results are expressed as means ± the standard error of the mean (SEM). Data statistical analyses were performed by one-way analysis of variance (ANOVA) using the GraphPad Prism 7.0 software (San Diego, CA 92108, USA). Differences were considered significant at *p* < 0.05.

## 5. Conclusions

For the first time, the neuroprotective properties of two originally synthesized myrtenal–adamantane conjugates were revealed in the rat dementia model. The new MAC- conjugates restored the impaired memory of demented rats via complex mechanisms. In both brain structures related to memory—the cortex and hippocampus—they decreased AChE activity, modulated monoamine (NA and 5-HT) levels, and exhibited antioxidant potential. The neuroprotective effects of MACs were stronger than those of natural myrtenal, which makes them potential therapeutic candidates for Alzheimer’s-type dementia.

## Figures and Tables

**Table 1:** The molecular descriptors and their corresponding Known Drug Indexes 2a and 2b (KDI_2a/2b_) for M and the MACs.

	RB	MW (g/mol)	HD	HA	Log *p*	PSA (Å^2^)	KDI_2a_	KDI_2b_
MAC-197	3	285.5	1	1.5	4.5	9.6	4.05	0.07
MAC-198	3	285.5	1	1	4.9	10.7	3.89	0.04
Myrtenal	1	150.2	0	2	1.8	36.6	3.38	0.01

**Table 2:** The binding affinities to the catalytic site of AChE as predicted by the scoring functions GoldScore (GS), ChemScore (CS), ChemPiecewise Linear Potential (ChemPLP), and Astex Statistical Potential (ASP) using GOLD (v5.4.1) (Cambridge, UK). HI6 is the co-crystallized ligand (4-(aminobarbonyl)-1-[({2-[(*E*)-(hydroxyimino)methyl]pyridiunum-1-yl}methoxy) methyl]pydidinium).

	ASP	ChemPLP	CS	GS
MAC-197	34.9	68.4	40.9	47.3
MAC-198	35.0	64.2	40.3	45.1
Myrtenal	23.8	44.0	28.5	35.1
HI6	48.1	85.6	29.1	62.4
Acetylcholine	24.1	43.1	18.2	36.6
Donepezil	59.1	83.2	43.3	56.9
Tacrine	42.1	66.0	35.1	57.1

## Data Availability

Not applicable.

## Supplementary Materials

The following supporting information can be downloaded at: https://www.mdpi.com/article/10.3390/molecules27175456/s1, Figure S1: ^1^H NMR spectrum of compound MAC-197; Figure S2: ^13^C NMR spectrum of compound MAC-197; Figure S3: ^1^H NMR spectrum of compound MAC-198; Figure S4: ^13^C NMR spectrum of compound MAC-198; Table S1: Definition of lead-like space, drug-like space, and Known Drug Space (KDS) in terms of molecular descriptors; Table S2: The 208 molecules with measured blood–brain barrier permeability (Log BB) and their corresponding molecular descriptors; Table S3: The known drugs that have an indication for the central nervous system and their corresponding KDI values.

## Sample Availability

Samples of the compounds are available from the authors.
