# Supplementary material for: New Myrtenal–Adamantane Conjugates Alleviate Alzheimer’s-Type Dementia in Rat Model
Source: Molecules. 2022 Aug 25;27(17):5456. doi: 10.3390/molecules27175456 (PMC9457974; doi:10.3390/molecules27175456)
Supplement: Supplementary file 1 [file molecules-27-05456-s001.zip › molecules-1863385-supplementary.pdf]

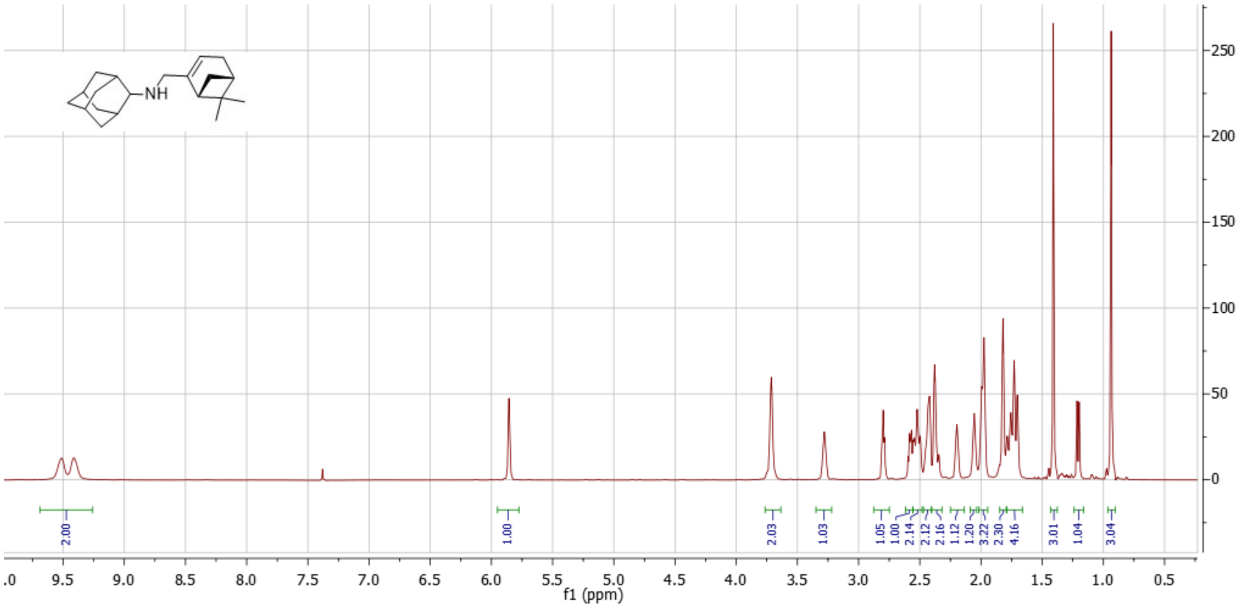

Figure S1. ^{1}H NMR spectrum of compound MAC-197

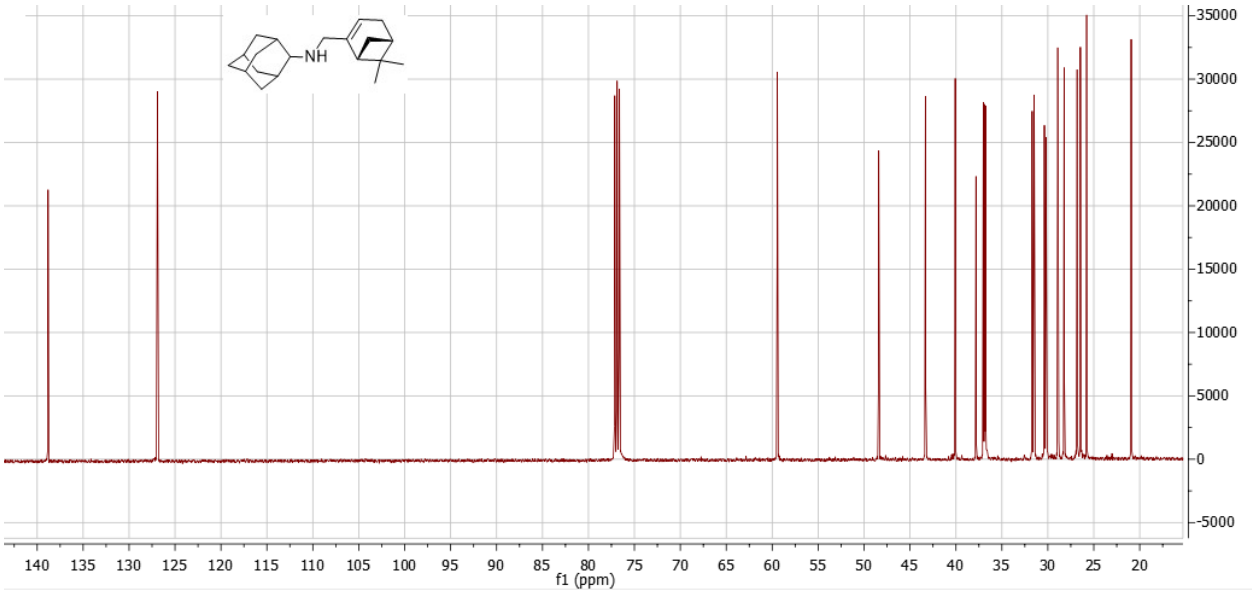

Figure S2. ^{13}C NMR spectrum of compound MAC-197

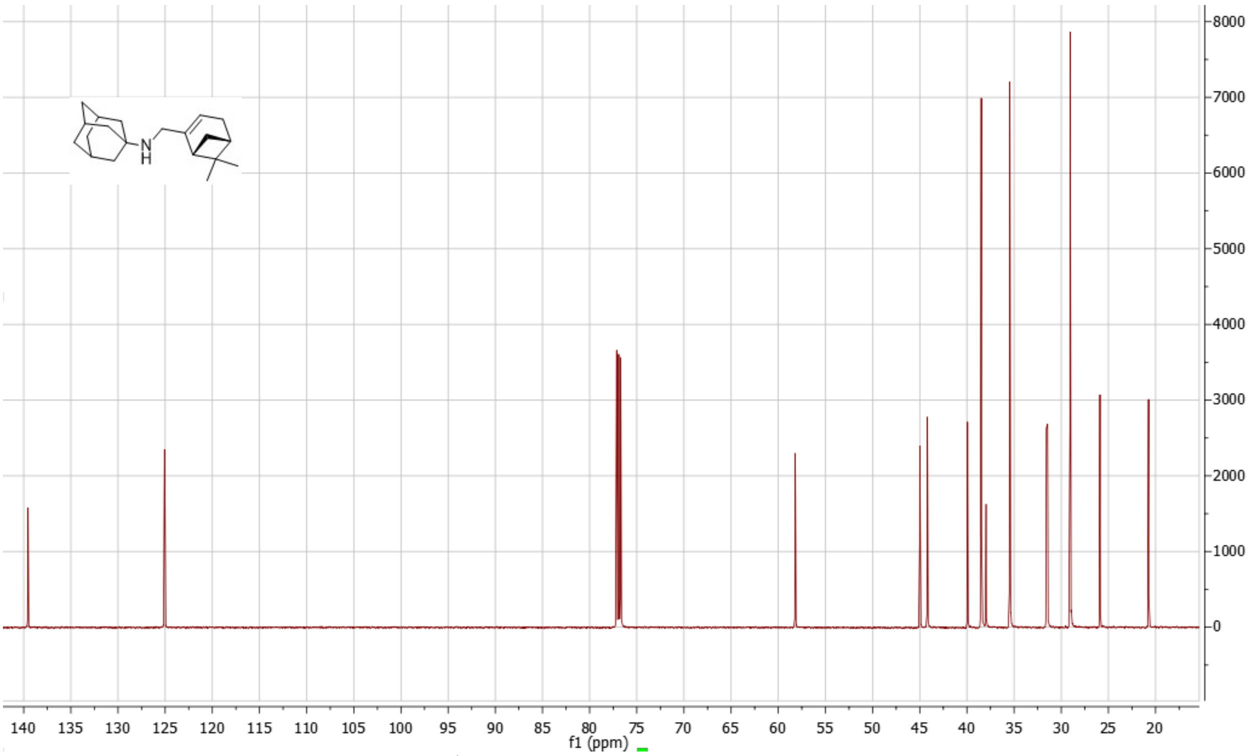

FigureS3. ^{1}H NMR spectrum of compound MAC-198

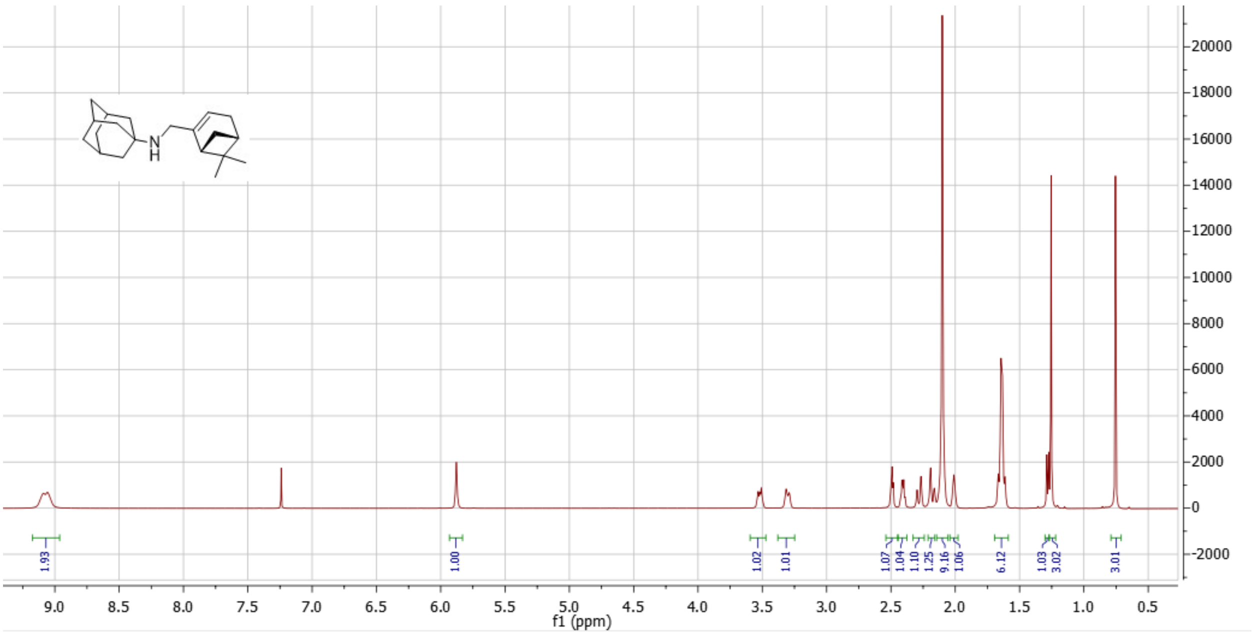

Figure S4. ^{13}C NMR spectrum of compound MAC-198

Table S1. Definition of lead-like, drug-like and Known Drug Space (KDS) in terms of molecular descriptors. The values given are the maxima for each descriptor for the volumes of chemical space used.

	**Lead-like Space**	**Drug-like Space**	**Known Drug Space**
Molecular weight (g mol^{-1})	300	500	800
Lipophilicity (Log P)	3	5	6.5
Hydrogen bond donors (HD)	3	5	7
Hydrogen bond acceptors (HA)	3	10	15
Polar surface area ($Å^2$) (PSA)	60	140	180
Rotatable bonds (RB)	3	10	17

Table S2. The 208 molecules with measured blood-brain-barrier permeability (Log BB) and their corresponding molecular descriptors.

Molecule	LogBB	RB	MW	HD	HA	LogP	PSA
1,1,1-Trichloroethane	0.40	0	133.4	0	0	2.4	0.0
1,1,1-trifluoro-2-chloroethane	0.08	0	118.5	0	0	2.3	0.0
1,2,3,4-Tetrahydroquinoline	0.70	0	133.2	1	1	2.2	13.7
1,2-dichloroethane	−0.14	0	99.0	0	0	1.5	0.0
1-Butanol	−0.02	3	74.1	1	1.7	0.9	23.3
1-hydroxymidazolam	−0.07	2	341.8	1	4.2	4.1	53.7
1-propanol	−0.16	2	60.1	1	1.7	0.3	23.3
2,2-dimethylbutane	1.04	1	86.2	0	0	3.6	0.0
2-bromopropane	0.56	0	123.0	0	0	1.6	0.0
2-methylpentane	0.97	2	86.2	0	0	3.6	0.0
2-Methylpropanol	−0.17	2	74.1	1	1.7	0.8	21.7
2-Propanol	−0.15	1	60.1	1	1.7	0.1	22.4
3-Methylhexane	0.90	3	100.2	0	0	4.0	0.0
3-Methylpentane	1.01	2	86.2	0	0	3.6	0.0
4-hydroxyalprazolam	−1.48	1	324.8	1	4.7	3.4	69.9
4-Hydroxymidazolam	−0.30	1	341.8	1	4.2	4.1	53.4
Acebutolol	−0.15	11	336.4	3	8.45	1.7	102.0
acetaminophen	−0.31	2	151.2	2	3.25	0.5	60.0
Acetone	−0.15	0	58.1	0	2	−0.2	30.5
Alovudine	−0.59	2	244.2	2	6.9	0.2	100.4
Alprazolam	0.02	0	308.8	0	3	4.4	48.8
Alprenolol	−0.23	9	249.4	2	3.95	3.1	41.8
Aminopyrine	0.00	1	231.3	0	5	1.0	32.9
amitriptyline	0.89	3	277.4	0	2	5.0	6.1
amobarbital	0.04	5	226.3	2	5.5	0.9	93.1
amphetamine	0.93	3	135.2	2	1	1.8	22.9
antipyrine	−0.10	0	188.2	0	4	0.4	33.4
Apaxifylline	−1.40	4	318.4	1	7	2.1	110.1
Aspirin	−0.61	2	180.2	1	4.5	1.2	81.8
Atenolol	−0.87	9	266.3	4	6.45	0.1	93.6
atropine	−0.06	5	289.4	0	4.7	1.9	60.2
Barbital	−0.14	2	184.2	2	4	0.6	100.6
Benzene	0.37	0	78.1	0	0	2.1	0.0
Benzocaine	0.27	3	165.2	1.5	3	1.9	61.2
Betahistine	−0.34	3	136.2	1	2.5	1.4	27.2
Bretazenil	−0.09	2	418.3	0	6.5	3.3	66.9
Bromocriptine	−1.10	6	654.6	2.25	10.75	3.5	114.5
Bromperidol	1.38	6	420.3	1	4.75	4.4	51.1
Bupropion	1.40	4	239.7	1	3	3.0	36.2
Buspirone	0.50	5	385.5	0	7.5	3.1	82.4
Butamben	0.42	5	193.2	1.5	3	2.2	61.2
Butanone	−0.08	1	72.1	0	2	0.3	27.8
Caffeine	−0.10	0	194.2	0	5	−0.1	72.4
Carbamazepine	−0.14	0	236.3	2	2	2.3	49.4

Carbon Disulfide	0.60	0	76.1	0	3	1.0	0.0
Carmustine	−0.52	5	214.1	1	4.5	1.9	73.3
Carteolol	0.01	7	292.4	3	5.95	1.4	77.6
Cefotetan	−1.89	9	575.6	2.25	12.5	0.7	240.3
Chlorambucil	−1.70	7	304.2	1	3	4.6	55.4
Chloroform	0.30	0	119.4	0	0	2.0	0.0
Chlorotrifluoroethane	−0.08	0	118.5	0	0	2.3	0.0
Chlorpromazine	1.06	4	318.9	0	2.5	5.1	10.0
Cimetidine	−1.42	7	252.3	3	5.5	0.4	101.0
Clobazam	0.40	0	300.7	0	6	2.1	55.6
Clonidine	0.10	2	230.1	2	2.5	1.6	31.9
Cocaine	0.60	3	303.4	0	6	2.3	58.1
Codeine	0.60	2	299.4	1	5.2	1.2	42.9
Cotinine	−0.22	0	176.2	0	4.5	0.3	43.8
Cyclohexane	0.90	0	84.2	0	0	2.9	0.0
Cyclopropane	0.00	0	42.1	0	0	2.1	0.0
Decane	0.67	7	142.3	0	0	6.1	0.0
Desflurane	0.11	1	168.0	0	0	2.1	8.9
Desipramine	1.20	4	266.4	1	2	4.8	18.6
Desmethylclobazam	0.36	0	286.7	1	5.5	1.9	66.5
Desmethyldiazepam	0.50	0	270.7	1	2	4.4	59.9
Desmonomethylpromazine	0.59	4	270.4	1	2	4.2	18.5
Diazepam	0.50	0	284.7	0	4	3.0	46.0
Dichloromethane	−0.10	0	84.9	0	0	1.4	0.0
Didanosine	−1.30	2	236.2	2	8.4	−0.5	101.6
Diethyl ether	0.00	2	74.1	0	1.7	0.9	8.8
Digoxin	−0.04	12	780.9	6	22.45	1.5	194.8
Diphenhydramine	1.26	6	255.4	0	3.7	3.3	13.5
divinyl ether	0.11	2	70.1	0	0.5	1.8	9.9
Domperidone	−0.80	4	425.9	2	6	3.9	98.5
Donepezil	0.89	6	379.5	0	5.5	4.4	42.2
Doxylamine	0.64	6	270.4	0	3.75	3.3	18.2
Enflurane	0.24	1	184.5	0	0	2.3	9.3
Enoxolone	−1.40	2	470.7	2	5.7	4.9	85.6
Ethanol	−0.16	1	46.1	1	1.7	−0.3	23.3
Ethenzamide	−0.05	3	165.2	2	3.25	0.9	58.0
Ethylbenzene	0.20	1	106.2	0	0	3.1	0.0
Etodolac	−1.42	4	287.4	2	2.75	3.9	60.8
etoposide	−2.00	7	588.6	3	16.95	0.6	162.4
Fentanyl	0.59	6	336.5	0	5	4.1	31.2
Fexofenadine	−0.98	12	501.7	3	6.45	3.9	87.8
Flumazenil	−0.30	2	303.3	0	6.5	1.9	76.8
flunitrazepam	0.06	1	313.3	0	5	2.3	87.6
Fluoromar	0.13	2	126.1	0	0	1.8	9.5
Fluoxetine	0.72	6	309.3	1	2.25	4.6	21.9
fluphenazine	1.51	7	437.5	1	6.2	4.3	38.5
Galanthamine	0.32	2	287.4	1	5.2	2.0	46.6
Gentisic acid	0.10	3	154.1	2	2.5	0.8	91.1
Granisetron	−0.69	2	312.4	1	5.5	2.5	55.5
haloperidol	1.34	0	197.4	1	4.76	2.3	49.6

Halothane	0.35	6	375.9	0	0	2.3	0.0
Heptane	0.81	4	100.2	0	0	4.7	0.0
Hexane	0.80	3	86.2	0	0	3.9	0.0
Hexobarbital	0.10	0	236.3	1	4	1.5	85.7
Hydroxyzine	0.39	9	374.9	1	7.4	2.8	43.5
Ibuprofen	−0.18	4	206.3	1	2	3.5	46.9
icotidine	−2.00	9	379.5	2	6.75	2.9	89.7
imipramine	1.06	4	280.4	0	2.5	4.4	10.0
indinavir	−0.75	14	613.8	4	13.9	2.6	110.8
indomethacin	−1.26	4	357.8	1	5.75	4.2	84.5
Iodophenazone	−0.10	0	314.1	0	4	1.6	33.3
Isoflurane	0.42	1	184.5	0	0	2.1	9.0
Lamotrigine	0.29	1	256.1	4	4	0.9	80.9
Levodopa	−0.80	6	197.2	5	4.5	−2.5	114.0
Levorphanol	0.00	1	257.4	1	2.75	2.9	28.5
Lidocaine	0.34	6	234.3	1	3	1.6	35.9
Loperamide	0.77	7	477.0	1	5.75	5.9	47.9
Lorazepam	0.44	1	321.2	2	3.7	3.2	78.1
Mannitol	−2.51	11	182.2	6	10.2	−3.1	124.1
Mefloquine	0.63	3	378.3	2	4.2	3.8	38.0
Methanol	0.02	0	32.0	1	1.7	−0.8	24.1
Mepyramine	0.49	7	285.4	0	4.25	3.3	27.9
Mesoridazine	−0.40	4	386.6	0	6.5	4.0	25.5
Methamphetamine	0.99	3	149.2	1	1.5	2.0	14.4
Methane	0.04	0	16.0	0	0	1.1	0.0
Methotrexate	−1.52	11	454.4	6.25	11.75	−1.8	228.6
Methoxyflurane	0.30	1	165.0	0	0	2.4	8.3
Methylcyclopentane	0.93	0	84.2	0	0	3.4	0.0
Metoprolol	1.15	10	267.4	2	5.65	1.9	51.7
Mianserin	0.99	0	264.4	0	3	3.3	10.0
Midazolam	0.40	0	325.8	0	2.5	5.2	32.6
Miloxacin	−0.92	2	263.2	0	6.7	0.3	106.2
Mirtazapine	0.50	0	265.4	0	3.5	3.4	17.3
Morphine	−0.20	2	285.3	2	5.2	0.9	60.7
Nalidixic acid	−0.66	2	232.2	0	4.5	1.6	86.6
Nevirapine	0.00	0	266.3	1	5	2.4	51.8
Nicotine	0.38	0	162.2	0	3.5	1.1	18.5
nitrogen	0.03	0	17.0	3	0.5	−2.0	43.1
nitrous oxide	0.03	0	44.0	0	4	−2.5	63.8
Northioridazine	0.80	4	356.5	1	2.5	5.4	17.9
Octane	0.69	5	114.2	0	0	4.8	0.0
Olanzapine	0.78	0	312.4	1	4	3.5	37.8
Omeprazole	−0.82	5	345.4	1	8	2.2	70.1
oxazepam	0.61	1	286.7	2	3.7	3.5	80.6
Oxirane	0.01	0	44.1	0	2	−0.5	13.7
Paliperidone	−0.67	4	426.5	1	9.2	2.5	91.2
Paraxanthine	0.10	0	180.2	1	5	0.0	86.6
Pentane	0.76	2	72.2	0	0	3.4	0.0
Pentazocine	0.51	3	285.4	1	2.75	3.6	27.2
Pentobarbital	0.10	4	226.3	2	4	2.1	95.9

Pentylenetetrazol	−0.03	0	138.2	0	3	0.5	50.5
Perchloroethylene	0.37	0	165.8	0	0	2.5	0.0
Pergolide	0.30	4	314.5	1	2.5	4.5	20.0
Pervertin	0.95	3	149.2	1	1.5	2.0	14.4
Phencyclidine	0.70	0	243.4	0	1	3.6	4.3
Phenothiazine	−0.48	0	199.3	1	0.5	3.5	12.7
Phenserine	1.00	2	337.4	1	6	2.5	50.4
Phenylbutazone	−0.50	3	308.4	0	5	4.1	53.0
Phenytoin	−0.04	2	252.3	2	3	2.5	59.3
Physostigmine	0.10	1	275.4	1	6	1.1	54.3
Pindolol	−0.14	7	248.3	3	3.95	1.8	51.5
Primidone	−0.07	1	218.3	2	2	3.0	73.0
Procaine	0.05	7	236.3	1.5	5	1.3	67.3
Promazine	1.23	4	284.4	0	2.5	4.7	10.0
Propranolol	0.64	7	259.3	2	3.95	3.1	41.5
Pseudocumene	0.16	0	120.2	0	0	3.3	0.0
Quinidine	−0.50	5	324.4	1	5.45	3.4	39.1
Ranitidine	−1.23	10	314.4	2	6	0.8	78.5
Ribavirin	−0.67	5	244.2	5	12.3	−2.5	154.9
Risocaine	0.55	4	179.2	1.5	3	1.8	61.2
Risperidone	0.00	3	410.5	0	7.5	3.2	69.9
Rivastigmine	0.88	4	250.3	0	5	2.1	41.3
Rolipram	0.61	3	275.3	1	4	2.2	61.7
Ropinirole	0.08	7	260.4	1	4.5	2.8	50.8
Salbutamol	−1.03	8	239.3	4	5.15	0.5	74.4
Salicylic acid	−1.10	2	138.1	1	1.75	2.3	68.4
Salicyluric acid	−0.44	4	195.2	1.25	3.5	1.3	104.9
Saquinavir	−0.95	14	670.9	5	13.7	2.5	179.0
Scopolamine	0.23	5	303.4	0	6.7	1.2	65.5
Sertraline	1.60	1	306.2	1	1.5	4.9	12.5
Sevoflurane	0.30	1	200.1	0	0	2.8	8.5
Sotalol	−0.28	7	272.4	3	7.7	0.2	83.7
Spiperone	0.26	6	395.5	1	6	4.2	66.3
Stavudine	−0.48	2	224.2	2	6.9	−0.1	100.4
Sulforidazine	0.18	4	402.6	0	6.5	3.9	48.7
Sulfur Hexafluoride	0.40	0	146.1	0	0.5	2.7	0.0
Tacrine	−0.10	1	198.3	2	1.5	2.6	33.2
Tamoxifen	0.92	9	371.5	0	2.75	6.3	14.8
Teflurane	0.27	0	180.9	0	0	2.3	0.0
Temelastine	−1.90	8	442.4	2	6	4.2	87.5
Terfenadine	0.64	11	471.7	2	4.45	6.5	38.1
Tretinoin	−0.49	9	300.4	1	2	5.3	49.3
Theobromine	−0.30	0	180.2	1	5	0.0	86.8
Theophylline	−0.29	0	180.2	1	5	0.0	87.1
Thiopental	−0.10	4	242.3	2	3.5	2.0	72.8
Thioperamide	−0.20	1	292.4	2	4	3.5	48.5
Thioridazine	0.20	4	370.6	0	3	5.9	9.5
Tiotidine	−0.80	9	312.4	6	7.5	0.1	159.0
Toliprolol	0.34	7	223.3	2	3.95	1.9	42.7
Toluene	0.37	0	92.1	0	0	2.7	0.0

Triazolam	0.74	0	343.2	0	3	5.0	46.9
Trichloroethene	0.34	0	131.4	0	0	2.4	0.0
Trifluoroperazine	1.40	0	133.1	0	1	1.9	12.4
Triflupromazine	1.44	4	352.4	0	2.5	5.5	10.1
Tripropylphosphine	1.02	6	160.2	0	0	4.9	0.0
Urea	−0.14	0	60.1	4	2	−2.2	83.7
Valproic acid	−0.22	5	144.2	1	2	2.7	43.9
Vinylbenzene	0.45	1	104.2	0	0	3.0	0.0
Vinyltrichloride	−0.10	0	133.4	0	0	2.5	0.0
Zalcitabine	−1.18	3	211.2	3	7.4	−0.4	98.4
Zanapezil	1.14	6	376.5	1	5	4.9	43.7
Zidovudine	−0.70	8	267.2	2	9.9	−1.2	165.3
Zolantidine	0.10	8	381.5	1	4.75	5.2	39.1
Zolpidem	−0.48	2	307.4	0	4.5	3.8	39.2

Table S3. The known drugs that have indication for the central nervous system and their corresponding KDI values.

Drug	KDI_{2a}	KDI_{2b}
Acamprosate	4.31	0.05
Acetaminophen or paracetamol	4.14	0.04
Adenosine	2.82	0.00
Alfentanil hydrochloride	4.05	0.04
Almotriptan	5.63	0.68
Alprazolam	4.21	0.10
Amantadine	3.45	0.01
Amitriptyline	3.60	0.04
Amisulpride	4.76	0.20
Amobarbital	5.13	0.32
Amoxapine	4.65	0.18
Amphetamine or dextroamphetamine	3.77	0.01
Aniracetam	4.39	0.12
Apomorphine	4.94	0.29
Aprepitant or fosaprepitant	4.87	0.20
Aripiprazole	5.14	0.38
Asenapine	3.62	0.04
Atomoxetine	4.34	0.12
Azasetron	5.18	0.39
Baclofen	4.12	0.06
Barbital	4.41	0.09
Bemegride or megimide	3.96	0.04
Benactyzine	5.04	0.29
Benperidol	5.80	0.81
Benzhexol or trihexyphenidyl	4.42	0.14
Benzphetamine	3.62	0.03
Benztropine or benzatropine	4.39	0.12
Biperiden	4.60	0.18
Bromazepam	4.99	0.28

Bromocriptine	3.92	0.01
Brotizolam	4.23	0.10
Buprenorphine	4.95	0.29
Bupropion	4.66	0.18
Buspirone	5.34	0.43
Butabarbital	4.91	0.23
Butorphanol	5.43	0.53
Butriptyline	3.81	0.05
Cabergoline	5.10	0.35
Caffeine	3.70	0.03
Cannabidiol	4.24	0.09
Carbamazepine	4.19	0.08
Carisoprodol	4.96	0.30
Carisoprodol	4.96	0.30
Citalopram	5.01	0.30
Chlordiazepoxide	4.53	0.14
Chlormezanone	3.11	0.01
Chlorphenesin	3.94	0.05
Chlorpromazine	4.57	0.09
Chlorzoxazone	4.29	0.11
Cisatracurium besilate	1.66	0.00
Clobazam	4.66	0.17
Clomipramine	4.07	0.07
Clonazepam	5.16	0.38
Clorazepate	4.67	0.19
Clozapine	4.73	0.20
Cocaine	5.21	0.38
Codeine	4.94	0.30
Cyclobenzaprine	3.73	0.04
Dalfampridine or Fampridine	3.47	0.01
Dantrolene	5.14	0.35
Desipramine or desmethylimipramine	4.15	0.09
Dexfenfluramine	4.10	0.06
Dexmedetomidine	4.01	0.06
Dextromethorphan	3.86	0.06
Dezocine	4.26	0.10
Diazepam	4.40	0.13
Dibenzepin	4.89	0.25
Diethylpropion	4.19	0.08
Dihydrocodeine	5.08	0.36
Dihydroergotamine	4.05	0.02
Dolasetron	5.14	0.35
Donepezil	5.00	0.30
Doxapram	5.19	0.36
Doxepin	4.03	0.08
Doxylamine	4.45	0.14
Droperidol	5.80	0.81
Dyclonine	4.56	0.17
Edaravone	3.52	0.02
Eletriptan	5.66	0.70

Eperisone	4.56	0.16
Ephedrine	4.34	0.07
Estazolam	4.22	0.10
Ethchlorvynol	3.84	0.01
Ethosuximide	3.88	0.03
Ezogabine or retigabine	5.28	0.43
Felbamate	4.10	0.08
Fentanyl citrate	4.87	0.25
Flunitrazepam	4.96	0.27
Fluoxetin or Fluoxetine	4.36	0.12
Flupenthixol or flupentixol	4.97	0.31
Fluphenazine	4.99	0.32
Flurazepam	5.24	0.39
Fluvoxamine	4.97	0.14
Frovatriptan	4.23	0.10
Gabapentin	3.82	0.02
Galantamine	5.13	0.38
Gamma Hydroxybutyric acid	4.19	0.02
Glutethimide	4.40	0.12
Granisetron	5.44	0.54
Halazepam	4.57	0.17
Haloperidol	5.40	0.52
Heroin or diamorphine	5.07	0.32
Hexobarbitone or hexobarbital	4.51	0.14
Hydromorphone	4.96	0.30
Hyoscine or scopolamine	5.06	0.32
Imipramine or melipramine	3.97	0.07
Isoflurane	2.92	0.00
Istradefylline	5.13	0.34
Ketamine	4.25	0.11
Ketazolam	4.85	0.21
Lacosamide	5.10	0.33
Lamotrigine	4.50	0.14
Levallorphan	4.71	0.21
Levetiracetam	4.31	0.04
Levodopa	3.16	0.00
Levorphanol	4.31	0.12
Lofepramine	4.34	0.12
Lofexidine	4.63	0.19
Lorazepam	5.50	0.55
Lorcaserin	3.44	0.02
Lormetazepam	5.41	0.50
Loxapine	4.34	0.11
Meclizine or meclozine	4.34	0.11
Melatonin	5.00	0.27
Memantine	3.59	0.02
Meperidine or pethidine	4.41	0.14
Mephenesin	4.65	0.13
Mephenytoin	4.54	0.14
Meprobamate	3.93	0.05

Mepyramine or pyrilamine	4.48	0.15
Mesoridazine	5.50	0.55
Methadone	4.48	0.16
Methamphetamine	3.69	0.02
Methocarbamol	4.69	0.19
Methohexital	5.32	0.45
Methotrimeprazine or Levomepromazine	4.33	0.12
Methoxyflurane	2.89	0.00
Methyldopa	3.29	0.00
Methsuximide or mesuximide	3.81	0.04
Methylphenidate or Dexmethylphenidate	3.79	0.05
Mianserin	3.65	0.04
Midazolam	3.79	0.05
Mirtazapine	3.87	0.06
Moclobemide	5.29	0.44
Modafinil	5.34	0.47
Morphine	5.10	0.35
Nabilone	4.77	0.22
Nalbuphine	5.70	0.72
Naloxone	5.73	0.75
Naltrexone	5.81	0.82
Naratriptan	5.84	0.85
Nefopam	3.79	0.05
Nicergoline	4.85	0.24
Nitrazepam	4.96	0.30
Nortriptyline	3.97	0.06
Olanzapine	4.68	0.19
Ondansetron	4.51	0.17
Oxazepam	5.32	0.45
Oxcarbazepine	4.61	0.16
Oxycodone	5.35	0.49
Oxymorphone	5.27	0.43
Paliperidone	5.21	0.36
Palonosetron	4.41	0.12
Paraldehyde	3.11	0.01
Paroxetine	5.13	0.38
Pemoline	4.29	0.06
Pentazocine	4.66	0.20
Pentobarbital	5.18	0.33
Perampanel	5.24	0.40
Pergolide	4.56	0.16
Perphenazine	5.14	0.38
Phendimetrazine	3.39	0.02
Phenobarbital	4.69	0.19
Phenprobamate	4.57	0.10
Phentermine	3.80	0.02
Phenytoin or fosphenytoin	4.75	0.19
Prazepam	4.70	0.22
Protriptyline	4.06	0.07

Pregabalin	3.68	0.01
Primidone	4.37	0.10
Prochlorperazine	4.59	0.15
Promazine	3.97	0.07
Promethazine	3.81	0.05
Propiomazine	4.98	0.29
Propofol	3.74	0.02
Propoxyphene	4.35	0.13
Quazepam	3.90	0.06
Quetiapine	5.52	0.60
Ramosetron	4.97	0.31
Rasagiline	3.89	0.03
Reboxetine	5.24	0.43
Remifentanil	4.60	0.17
Remoxipride	5.61	0.65
Reserpine	3.38	0.01
Riluzole	4.49	0.13
Risperidone	5.19	0.37
Rivastigmine	4.78	0.21
Rizatriptan	5.34	0.47
Rocuronium	4.28	0.08
Ropinirole	5.02	0.32
Rotigotine	4.41	0.14
Rufinamide	5.06	0.30
Secobarbital	5.17	0.35
Selegiline	3.75	0.03
Sertraline	3.80	0.05
Sevoflurane	3.03	0.01
Sibutramine	3.83	0.05
Stiripentol	4.76	0.21
Succinylcholine	4.50	0.14
Sufentanil	4.65	0.19
Sumatriptan	5.44	0.54
Tacrine	4.03	0.06
Tapentadol	4.41	0.12
Temazepam	5.28	0.43
Tetrabenazine	5.22	0.38
Thiethylperazine	4.59	0.16
Thiopental	5.16	0.35
Thioridazine	3.94	0.05
Thiothixene	4.79	0.23
Tiagabine	5.50	0.59
Tizanidine	5.11	0.35
Tolcapone	5.22	0.41
Tolperisone	4.50	0.14
Topiramate	5.03	0.32
Tramadol	4.84	0.25
Trazodone	5.35	0.44
Triazolam	4.22	0.10
Triflupromazine	3.93	0.05

Trimethobenzamide	4.85	0.18
Tropisetron	4.54	0.17
Troxidone or trimethadione or tridione or trimethin or Trimetadione or minoaleviatin	3.44	0.01
Valproate or valproic acid	4.25	0.04
Valpromide	4.14	0.04
Venlafaxine	5.04	0.32
Vigabatrin	3.65	0.01
Vilazodone	5.07	0.36
Viloxazine	4.92	0.26
Vortioxetine	4.35	0.13
Zaleplon	5.24	0.39
Ziprasidone	5.61	0.66
Zolmitriptan	5.62	0.65
Zolpidem	4.80	0.24
Zonisamide	4.64	0.13
Zopiclone or eszopiclone	3.85	0.04
Zotepine	4.14	0.08
KDI AVERAGES CNS DRUGS	4.54	0.21
SD CNS DRUGS	0.67	0.19
